# Effects of Yellow Light on Airborne Microbial Composition and on the Transcriptome of Typical Marker Strain in Ward

**DOI:** 10.1155/2022/8762936

**Published:** 2022-05-18

**Authors:** Xuanqi Zhao, Jing Wei, Wenjie Chen, Xuan Xu, Ruizhe Zhu, Puyuan Tian, Tingtao Chen

**Affiliations:** ^1^School of Life Science, Nanchang University, Nanchang, China; ^2^National Engineering Research Center for Bioengineering Drugs And The Technologies, Institute of Translational Medicine, Nanchang University, Nanchang, China; ^3^Queen Mary School, Nanchang University, Nanchang, China; ^4^Huankui Academy, Nanchang University, Nanchang, China; ^5^Precision Medicine Institute, The First Affiliated Hospital, Sun Yat-Sen University, Guangdong, China

## Abstract

Airborne diseases are transmitted by pathogens in the air. The complex microbial environment in wards is usually considered a major cause of nosocomial infection of various diseases which greatly influences the health of patients with chronic diseases, whereas the illuminant of wards impacts on the microbe especially the disease marker strain is seldom studied. In the present study, high-throughput sequencing was used to study the effect of yellow light on airborne microbial composition, and changes of transcriptome of marker strains *Escherichia coli*, *Staphylococcus aureus* and *Pseudomonas aeruginosa*, which were isolated from wards, were further studied after the irradiation by yellow light. High-throughput sequencing results indicated that yellow light significantly decreased *α*-diversity. The relative abundance of Firmicutes at the phylum level, and *Clostridium sensu strict*o *1*, *Paraclostridium* at the genus level were significantly reduced. RNA sequencing results declared that yellow light significantly downregulated the genes associated with flagella, heme transport system and carbohydrate, amino acid metabolism in *E. coli*, and the genes related to arginine biosynthesis and the biosynthesis of isoleucine, leucine, and valine in *S. aureus*. Meanwhile, yellow light significantly upregulated the genes relating to porphyrin metabolism *in P. aeruginosa*. In conclusion, our work reveals the impacts of yellow light on the microbe in wards, pointing out the application value of yellow light in the prevention of infectious diseases in clinical practice.

## 1. Introduction

Hospital environment including hospital and healthcare settings [1] has a pivotal impact on patients to meet them with a variety of needs, such as medical care and spiritual comfort [2, 3]. Within the hospital, the indoor air contains suspended particles of multifarious properties and mediates the short-distance and long-distance transmission of microbes made up of bacteria and fungi [4]. Studies have shown that the spreading of microbes is connected with microbial pollution and diseases [5–7]. Thereinto, Proteobacteria, Phylum Firmicutes are wildly distributed in hospital wards and *Escherichia coli*, *Pseudomonas aeruginosa*, and *Staphylococcus aureus* are the most common pathogenetic bacteria in nosocomial acquired infections which mainly transmits in the manner of hospital air [8–13]. The spreading of these bacteria widely in the air causes hospital-acquired infections and occupational diseases [14, 15], affecting children, aged people, chronic patients with a weak immune system, and healthcare workers [16]. Obviously, the increasing in the number or diversity of pathogens will certainly raise the risk of disease. Thus, the risk of infection from airborne pathogens is an important consideration that must be taken into account on hospital wards for individuals or groups [17].

In recent years, visible light has been shown to have multifarious effects on bacteria [18]. There were significant differences in the composition, abundance, and viability of microbial communities associated with household dust when exposed to sunlight [19]. For these three representative vital pathogenic bacteria of nosocomial infection, many studies have revealed the inactivation effect of visible light. For example, McClary and Boehm irradiated *S. aureus* with full spectrum light 6 hours and measured transcript abundance by RNA sequencing to find out that *S. aureus* was inhibited by full spectrum sunlight [20]. Exposing *S. aureus*, *P. aeruginosa*, and *E. coli* strains to a single blue laser irradiation (450 nm) and counting the number of living bacteria, De Sousa et al. concluded that blue light significantly inhibited the growth of bacteria under low-intensity conditions [21]. Additionally, the effect of yellow light on bacteria has been preliminarily explored. Among them, Weng et al. injected *S. aureus* to construct infected wounds in mice then treat it with yellow light and a ZnO composite, thereby achieving effective sterilization [22]. Zhao et al. utilized yellow light LED and MG1363-pMG36e-mCXCL12 in the treatment of the burn wounds in mice and found that skin pathogens were significantly reduced, excessive inflammatory response was inhibited, and the wound healing was promoted [23].

Currently, there is still no research to evaluate the effect of yellow light on wards microorganisms. There are also few studies on the effect of yellow light on *E. coli*, *S. aureus*, and *P. aeruginosa* at transcriptome level. Hence, in the present study, we first performed high-throughput sequencing method to preliminarily identify the distribution and composition of common microorganisms on the yellow light-treated wards. In order to explore the effect of yellow light on specific pathogenic bacteria in wards, we selected 3 bacteria (including Gram-positive and Gram-negative bacteria) that were highly related with nosocomial infection. These pathogenic bacteria were detected by a viable count method. Meanwhile, we explored the effects of yellow light on *E. coli*, *S. aureus*, and *P. aeruginosa* by conducting the transcriptome analysis of these pathogens. These studies will help to reveal the effect of yellow light on the microorganism in the air of hospital wards and the transcription level of related pathogenic bacteria. This study provides a theoretical basis for the effective control methods of the common pathogenic microorganisms, guiding the prevention of ward infectious diseases, which is of great significance to reduce the nosocomial infection and transmission.

## 2. Materials and Methods

### 2.1. Air Sample Collection and Processing

The study was performed in June 2019 (summer; *T* = 30.1°C; RH = 43.8%) in the second affiliated hospital of Nanchang University, Nanchang, China. Analyses were conducted in general ward of gastrointestinal surgery department. The wards are located on the eighth floor of the hospital. Each ward has a door for regular entrance, and the rooms are equipped with natural ventilation with windows. The light treatment of wards consisted of a light-emitting diode (LED) yellow light (570–600 nm, 16 W) or light-emitting diode (LED) white light (all-spectrum, 16 W) for 15 hours a day for 2 months.

Air sampling of each ward was done by impaction using a small flow TSP air sampler (MAS100, Merck KGaA, Darmstadt, Germany) with a flow rate of 10 L/min. Air samples were collected using glass fiber filter sampling membrane with 0.22 *μ*m pore size, and the sampling time is 72 h. Air sampling was performed at a height of 1.0 m above the floor to simulate the breathing zone. After removing the sampling membrane, we wrap it with tin foil and store at -80°C.

### 2.2. Acquisition and Treatment of Bacterial Strains

Strains of *E. coli* ATCC 25922, *S. aureus* ATCC 25923, and *P. aeruginosa* ATCC 27853 were obtained from the American Type Culture Collection (ATCC) and were provided by the clinical laboratory of the second affiliated hospital of Nanchang University.

The above strains were grown in LB overnight and then subcultured into fresh medium and grown at 37°C with aeration to an OD_600_ of 1.0. 100 *μ*L bacterial cultures were kept on Petri plates containing Luria-Bertani culture medium (BD, New Jersey, PA) and irradiating with LED yellow light or white light for different durations (8 h, 12 h, 20 h, and 24 h for each group, respectively). After irradiation, the bacteria were incubated at a temperature of 37°C until stable growth period. Then, the standard plate count method was used to determine the total number of viable cells of *E. coli*, *S. aureus*, and *P. aeruginosa* as the colony-forming units (CFU) on nutrient agar media after incubation at 37°C for 24 h.

Each bacterial cultures were irradiating with LED yellow light or white light for different durations (4 h for *E. coli* and *S. aureus*, 8 h for *P. aeruginosa*). All the strains were collected in Eppendorf tube (Trypticase Soy Broth, BD, New Jersey, PA), placed in liquid nitrogen (30 min) for quick freezing, and then stored at -80°C.

### 2.3. High-Throughput Sequencing and Data Analysis

Bioaerosol total genomic DNA was extracted from about the glass fiber filters using TIANamp Bacteria DNA Kit (QIAGEN) following the manufacturer's instructions. Extracted DNA was conducted whole genome sequencing in Shanghai Personal Biotechnology Cp. Ltd.

The concentration and quality of extracted genomic DNA were tested prior to sequencing using the NanoDrop spectrophotometer (Thermo, United States). Primers of 338F/806R were used to amplify the V3-V4 region of 16S rRNA genes of the extracted genomic DNA. FLASH was applied to merge overlapped reads, and sequence analysis was done using UPARSE software package. Reads with quality scores lower than 20, ambiguous bases, and improper primers were discarded before clustering. Simultaneously, chimaeras were checked and eliminated during clustering. The resultant high-quality sequences were clustered into operational taxonomic units (OTUs) at 97% similarity. According to the annotation of taxa, the relative abundance of total bacteria in each sample was categorized to different classification levels (Kingdom, Phylum, Class, Order, Family, and Genera). The alpha diversity index (Shannon index and Chao 1 index) was calculated, and the beta diversity index (principal component analysis) was mapped in QIIME2 software (version 2019.4). The raw reads were deposited in the Sequence Read Archive (SRA) database of NCBI (PRJNA778878).

### 2.4. RNA Sequencing and Data Analysis

Total bacterial RNA was extracted from strains of *E. coli*, *S. aureus*, and *P. aeruginosa* under different light treatments using the TRIzol reagent (Thermo Fisher Scientific, USA). DNase I was added to eliminate potential contaminating genomic DNA. The quality of RNA was verified using a NanoDrop spectrometer (Thermo, United States). The A_260/280_ absorbance ration was in the range of 2.0-2.2 and the A_260/230_ ratio was above 1.8. Ribosomal RNA was removed using the Ribo-Zero rRNA Removal Kit (Illumina, San Diego, CA, USA). Then, the mRNA was sequenced and the enriched mRNA was broken into short fragments and reverse transcribed into cDNA afterwards. When the second strand of cDNA was synthesized, the using of dUTP improves the accuracy of the results. The DNA library was sequenced with pair-end sequencing (2 × 150 bp) on the Illumina Hiseq platform by Shanghai Personal Biotechnology Cp. Ltd. The transcriptome data were deposited in the Sequence Read Archive (SRA) under the accession number: PRJNA779215.

Raw RNA-seq data was checked by FastQC software (http://www.bioinformatics.babraham.ac.uk/projects/fastqc/) to realize the data filter and quality control analysis. We used the fastx-toolkit to remove potential adapter sequences (http://hannonlab.cshl.edu/fastx_toolkit/index.html). The remaining clean reads were mapped to corresponding bacterial genome using Bowtie2 software (http://bowtie-bio.sourceforge.net/index.shtml).

The RPKM (reads per kilobase per million reads) values were used to measure the expression level of each gene in the sample. The differential expression of the transcripts was calculated using DESeq software (version 1.18.0). The differentially expressed genes (DEGs) were screened according to the differences in expression and the *p* values. The genes with *p* < 0.05 and an absolute value of log_2_ fold change > 1 were defined as displaying a significant expression difference. The volcano map of differentially expressed genes were plotted using ggplots2 software package on R language.

Then, we use TopGO for GO enrichment analysis. By the calculation of the gene number in each term and the calculation of *p* value by hypergeometric distribution method, we find the GO term with a significant enrichment of differential genes. We counted the number of differentially expressed genes at different levels of each KEGG pathway to determine the metabolic pathways and signaling pathways that differentially expressed genes were mainly involved in.

Finally, novel transcripts were predicted using the Rockhopper program (http://cs.wellesley.edu/~btjaden/Rockhopper) comparing against the NCBI nonredundant database, Swiss-Prot database, and eggNOG database.

### 2.5. Statistical Analysis

Statistical analysis was carried out using Student's *t*-test by SPSS 26.0 and GraphPad Prism 8.0 software. All data are presented as mean ± standard deviation. Statistical analysis was performed using the Student *t*-test, and all *p* values < 0.05 were considered significant.

## 3. Results

### 3.1. The Illumination of Yellow Light Influenced the Composition of the Microbiome

High-throughput sequencing analysis was used to monitor the differences of airborne microorganism in the hospital under different light modes. All hospital airborne samples underwent sampling collection, DNA extraction, target fragment obtaining, and Illumina sequencing. In total, 2,492,655 effective original sequences were collected with an average of 276,961.67 reads per sample passing quality filtering. The valid data was filtered, and those sequences with over 97% similarity were clustered as one OTU, and a total of 851 unique OTUs were clustered with an average of 94.56 OTUs per sample.

**Figure 1:**
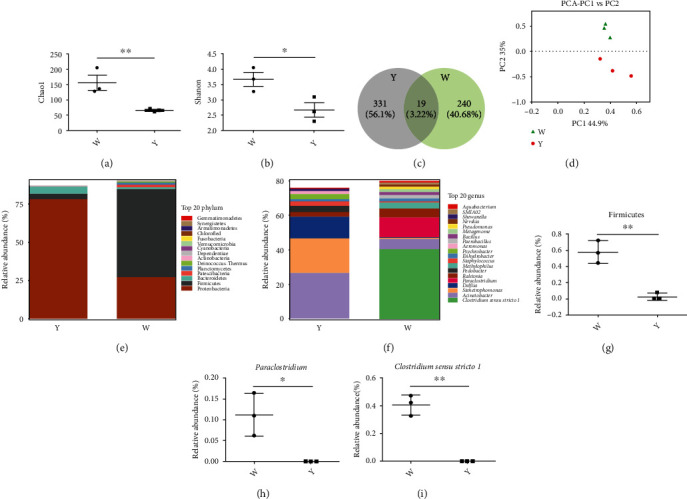
The effects of the irradiation of visible light (white light and yellow light) on the airborne microorganisms' diversity of medical facilities. (a, b) The Chao 1 index and Shannon index. (c) Scalar Venn representation. (d) Principal component analysis (PCA) results. (e) The relative abundance of the bacteria at phylum level. (f) The relative abundance of the bacteria at genus level. (g–i) The relative abundance of bacteria which are significantly changed. W: the airborne microorganisms under the radiation of white light; Y: the airborne microorganisms under the radiation of yellow light. ^∗^*p* < 0.05, ^∗∗^*p* < 0.01.

The abundance of dominant bacteria microorganisms in the nosocomial ward bioaerosols were identified by high-throughput sequencing. The sharp reduction of Chao 1 index and Shannon index in group Y indicated that the *α*-diversity in group Y had markedly reduced (*p* < 0.05) (Figures 1(a) and 1(b)). According to the results of the Venn diagram, there are 331 and 240 unique OTUs in group Y and group W, respectively, with a total of 19 core OTUs in both groups (Figure 1(c)). Beta diversity index focuses on differences between samples. Through principal component analysis (PCA), the two main components PC1 and PC2 of the genetic diversity of the microbial community structure represent 44.9% and 35% of the total variable, PCA indicated that all samples in group Y were obviously departed from group W, and all samples in group W clustered together (Figure 1(d)).

The relative abundance of the top twenty microorganism populations indicated that Proteobacteria, Firmicutes, and Bacteroidetes constituted the three most dominant phylum both in the Y and W groups. Proteobacteria and Firmicutes were the two most prevalent microbes among the hospital air microorganisms, and their total percentage accounted for approximately 85% and 82% in the W group and Y group, respectively (Figure 1(e)). Compared with group W, the relative abundance of Firmicutes was significantly decreased (Figure 1(g)). At the genus level, dominant microorganisms were *Clostridium sensu stricto 1* (relative abundance greater than 40.5%), *Paraclostridium* (relative abundance greater than 11.2%), *Acinetobacter* (relative abundance greater than 5.9%), and *Ralstonia* (relative abundance greater than 5.49%) in W group (Figure 1(f)), while *Clostridium sensu stricto* 1 and *Paraclostridium* had not detected in group Y (Figures 1(h) and 1(i)).

### 3.2. The Illumination of Yellow Light Affects the Number of E. coli, P. aeruginosa, and S. aureus

**Figure 2:**
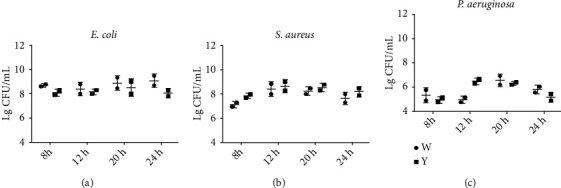
The effects of the irradiation of visible light (white light and yellow light) on colony forming unit of *E. coli*, *S. aureus*, and *P. aeruginosa*.

As represented in Figure 2, the concentrations of bacteria varied and ranged from 10^3^ CFU/mL to 10^10^ CFU/mL. After the yellow light treatment, the concentration of *E. coli* and *P. aeruginosa* was decreased, while *S. aureus* concentration was no reduced (Figure 2).

### 3.3. Changes in Gene Expression of E. coli Caused by Yellow Light Irradiation

**Figure 3:**
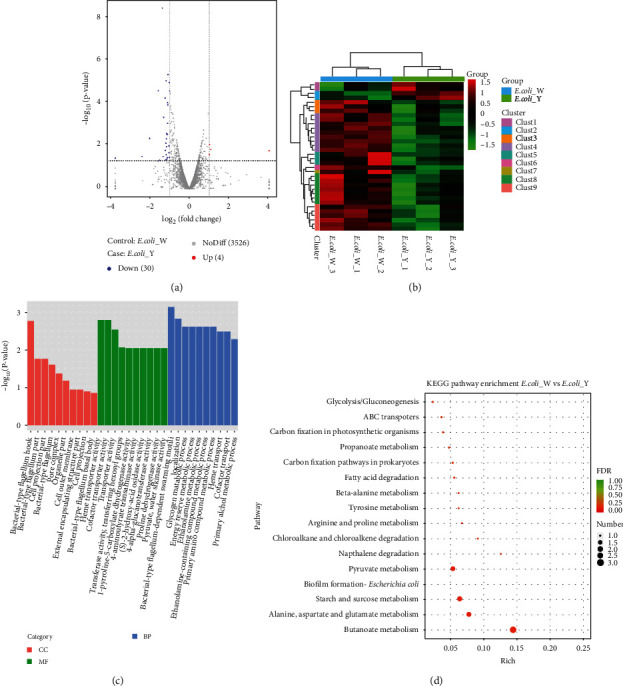
Global comparison of transcript profiles and DEGs in *E. coli* W vs. *E. coli* Y. (a) Volcano plots showing transcriptional differences in pairwise comparisons of transcriptomes. (b) Heat map of the hierarchical cluster analysis of gene expression. (c) GO term enrichment results. (d) KEGG pathway enrichment analysis of differentially expressed genes in response to yellow laser. *E. coli*_W: *E. coli* under the radiation of white light; *E. coli*_Y: *E. coli* under the radiation of yellow light.

By using the Illumina sequencing platform, 47,219,278 original sequences and 45,624,814 original sequences were harvested from *E. coli* W and *E. coli* Y, respectively, including 44,152,460 and 43,044,670 clean reads after strict filtration. After the DEG screening from the collection of mapped genes, there were 34 DEGs found, including 4 upregulated and 30 downregulated (Figure 3(a)). Hierarchical clustering of gene expression patterns was performed by analyzing the RPKM expression values (Figure 3(b)).

In the *E. coli* Y vs. *E. coli* W, 492 DEGs were annotated in biological process, 303 DEGs were annotated in molecular function, and 153 DEGs were annotated in cellular component. Then, we selected the top 10 GO term entries with the most significant enrichment for display (*p* value < 0.05). Comparing *E. coli* Y to *E. coli* W, in BP category (bacterial-type flagellum-dependent swarming motility, locomotion, and glycogen metabolic process), CC category (bacterial-type flagellum hook, bacterial-type flagellum part, and cell projection part), and MF category (heme transporter activity, cofactor transporter activity, and transporter activity), the top 3 significantly changed subcategories were listed, respectively (Figure 3(c)).

Subsequently, KEGG metabolic pathway variance analyses were performed and the DEGs in *E. coli* Y vs. *E. coli* W were matched to 16 different KEGG pathways. The most significantly changed pathways included butanoate metabolism, alanine, aspartate and glutamate metabolism, starch and sucrose metabolism, and biofilm formation-*E. coli* and pyruvate metabolism (*p* value < 0.05) (Figure 3(d)).

### 3.4. Changes in Gene Expression of S. aureus Caused by Yellow Light Irradiation

**Figure 4:**
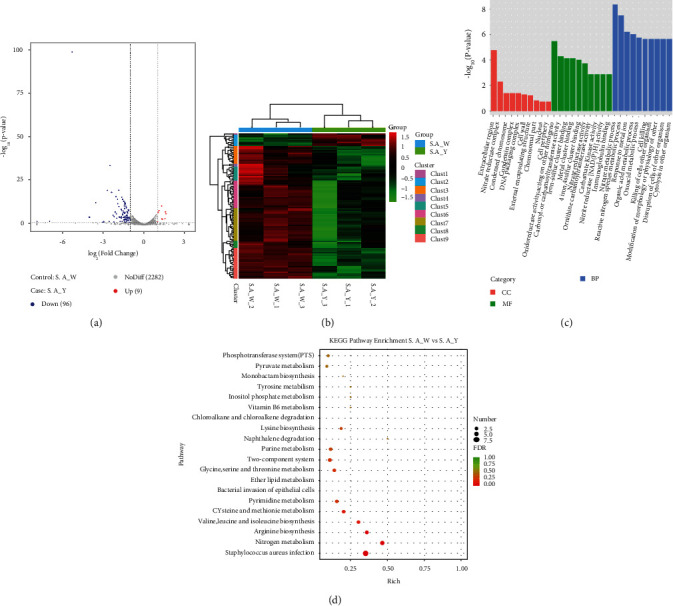
Global comparison of transcript profiles and DEGs in *S. aureus* W and *S. aureus* Y. (a) Volcano plots showing transcriptional differences in pairwise comparisons of transcriptomes. (b) Heat map of the hierarchical cluster analysis of gene expression. (c) GO term enrichment results. (d) KEGG pathway enrichment analysis of differentially expressed genes in response to yellow laser. *S. A*_W: *S. aureus* under the radiation of white light; *S. A*_Y: *S. aureus* under the radiation of yellow light.

*S. aureus* W and *S. aureus* Y contained a total of 41,032,965 and 41,927,208 reads, respectively, 38,015,275 and 39,241,966 clean reads involved correspondingly. A total of 105 genes were identified differentially expressed in the comparison of *S. aureus* Y and *S. aureus* W, with 9 upregulated and 96 downregulated (Figure 4(a)). Hierarchical clustering was performed to determine the unknown function of genes (Figure 4(b)).

In the *S. aureus* Y vs. *S. aureus* W, 1519, 685 and 281 DEGs were annotated in biological process, molecular function, and cellular component, respectively. Nitrate metabolic process, reactive nitrogen species metabolic process, and response to metal ion were top 3 significantly changed subcategories in BP category; extracellular region, nitrate reductase complex, and condensed chromosome were in CC category; oxidoreductase activity, acting on other nitrogenous compounds as donors, carboxyl- or carbamoyltransferase activity, and iron-sulfur cluster binding were in MF category (Figure 4(c)).

There were 40 different KEGG pathways found in *S. aureus* Y vs. *S. aureus* W, including Staphylococcus aureus infection, nitrogen metabolism, arginine biosynthesis, valine, leucine and isoleucine biosynthesis, pyrimidine metabolism, bacterial invasion of epithelial cells, and ether lipid metabolism (*p* value < 0.05) (Figure 4(d)).

### 3.5. Changes in Gene Expression of P. aeruginosa Caused by Yellow Light Irradiation

**Figure 5:**
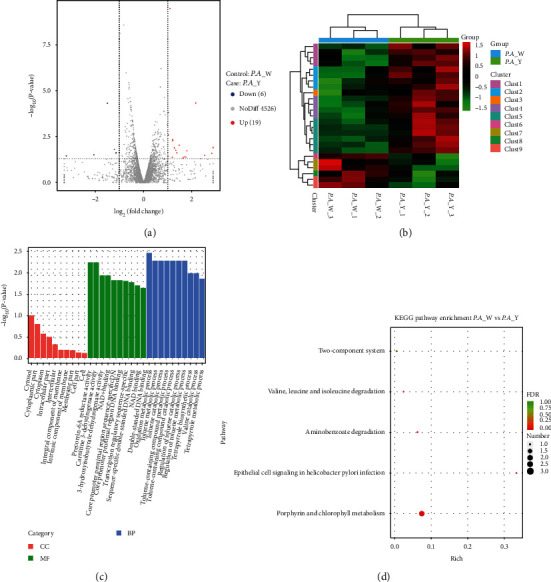
Global comparison of transcript profiles and DEGs in *P. aeruginosa* W vs. *P. aeruginosa* Y. (a) Volcano plots showing transcriptional differences in pairwise comparisons of transcriptomes. (b) Heat map of the hierarchical cluster analysis of gene expression. (c) GO term enrichment results. (d) KEGG pathway enrichment analysis of differentially expressed genes in response to yellow laser. *P. A*_W: *P. aeruginosa* under the radiation of white light; *P. A*_Y: *P. aeruginosa* under the radiation of yellow light.

In *P. aeruginosa* W and *P. aeruginosa* Y, a total of 50,340,184 and 49,181,358 raw reads was detected, respectively, containing 46,444,907 and 46,098,015 clean reads correspondingly. 25 DEGs were detected between the comparison of *P. aeruginosa* Y and *P. aeruginosa* W; thereinto, 19 were up-regulated and 6 were repressed (Figure 5(a)). We performed a hierarchical clustering analysis, and the results are shown in Figure 5(b).

In the *P. aeruginosa* Y vs. *P. aeruginosa* W, 391 DEGs were annotated in biological process (BP) GO terms; among them, the top 3 significantly changed subcategories were oxidation-reduction process, toluene metabolic process, and toluene catabolic process. There were 41 DEGs annotated in cellular component GO terms, and cytosol, cytoplasmic part, and cytoplasm were changed significantly in CCs. 174 DEGs annotated in molecular function (MF) GO terms, precorrin-6A reductase activity, carnitine 3-dehydrogenase activity, and 3-hydroxyisobutyrate dehydrogenase activity were the top 3 significantly changed subcategories (Figure 5(c)).

The DEGs in *P. aeruginosa* Y vs. *P. aeruginosa* W were matched to 5 different KEGG pathways. The most significantly changed pathways were porphyrin and chlorophyll metabolism (*p* value < 0.05) (Figure 5(d)).

## 4. Discussion

This study characterized the differences in the composition of air microflora and the differences in the transcriptomic expression of *E. coli*, *P. aeruginosa*, and *S. aureus* under yellow and white light, with the aim of determining whether the yellow light had an impact on some of common pathogenic bacteria in nosocomial acquired infections.

Our results suggested that the exposure to yellow light at 570–600 nm in hospital wards had relationships with the structure, composition, and functional pathways of atmospheric microbiota. After treatment with yellow light, the community diversity and the bacterial load in the air were decreased and the sanitation of hospital wards were improved. Among the decreased strains, Firmicutes consisted of a kind of typical pathogenic bacteria such as *Bacilli* and *Clostridia* causing nosocomial infection, including inflammation, emesis, and diarrhea [24–27]. To be specific, at the genus level, the relative abundance of *Clostridium sensu stricto 1* and *Paraclostridium* decreased significantly. *Paraclostridium*, found in the human gut, had the ability to exacerbate inflammation and has been shown to be associated with diseases such as metastatic osteomyelitis, necrotizing pneumonia, and bacteremia [28]. As obligate anaerobe, *Clostridium sensu stricto 1* formed air-tolerant, metabolically dormant spores to transmit the disease [29]. When the main cause of nosocomial infection is the presence of pathogens [7], our treatment showed a certain ability of disinfection to modulate the air quality of wards and reduce the occurrence of nosocomial infection.

Previous high-throughput sequencing results showed that yellow light decreased the relative abundance of airborne pathogens. We selected three common pathogenic bacteria from hospital. *P. aeruginosa* and *E. coli* were the most prevalent Gram-negative conditional pathogens in nosocomial infections [11]. The Gram-positive bacterium *S. aureus* was a well-known opportunistic pathogen, which was considered one of the leading causes of hospital-acquired infections [30]. We further studied the effect of yellow light on the above three pathogenic bacteria through viable count method and RNA-Seq.

After yellow LED illumination at 570–600 nm, the number of *E. coli* decreased by the viable count method, and we were surprised to find that the microbial gene associated with carbohydrate, amino acid metabolism had lower expression amount. This indicated that irradiation with yellow light had an impact on microbial (*E. coli*) nutrient metabolism. Downregulation of carbohydrate metabolism and amino acid metabolism genes closely related to microbial carbon and nitrogen source decomposition and utilization which weakened the energy harvesting and restrained the bacteria growth [31, 32]. Furthermore, the products of the above metabolisms, such as short-chain/branched-chain fatty acids and biogenic amines, might mediate the growth of *E. coli* [33]. The expression of flagella-related genes was significantly reduced after the yellow light illumination, resulting in the weakening of virulence and pathogenicity [34–36]. In addition, we found that the expression of heme transport system-related genes was reduced in *E. coli*. The decrease expression of the gene, which was closely related to the ability of bacteria to cause diseases, led to the reduction of the virulence of *E. coli* [37, 38]. In conclusion, LED yellow light inhibits the growth of *E. coli* and reduces the pathogenicity by inhibiting the energy metabolism and the metabolic synthesis of important growth substances and downregulating genes related to flagella and heme transport system.

After yellow LED illumination, the number of *P. aeruginosa* decreased by the viable count method; afterwards, we observed that yellow light significantly upregulated the genes relating with porphyrin metabolism. One possible explanation for the phenomenon was the difference in the number of endogenous porphyrin compounds in the bacterial cells. When bacterial cells were exposed to the energy of light, photosensitizers would be excited, such as either exogenous or endogenous porphyrin molecules [39]. Once these porphyrin compounds absorbed visible light in the presence of oxygen, reactive oxygen species (ROS) were produced, consequently poisoning the bacterial [39]. The ROS such as singlet oxygen, superoxide anion, and the hydroxyl radical might damage membrane lipids, enzymes, proteins, or DNA [40]. Therefore, the upregulation of porphyrin metabolism-related genes in *P. aeruginosa* caused by yellow light can increase the production of reactive oxygen species and promoting the bacterial death.

Though the number of *S. aureus* did not decrease after yellow light irradiation, the metabolic pathways of *S. aureus* were significantly affected and the pathogenicity decreased. Arginine biosynthesis in *S. aureus* was pivotal in diseases leading. The biosynthesis of arginine, which was then metabolized to synthesize ammonia, to alleviate the influence of ROS [41]. Additionally, the downstream products of arginine production, such as ornithine and polyamines, were shown to be pivotal compound in cell metabolism [42]. Hence, downregulation of arginine biosynthesis limits the ability of *S. aureus* easing ROS killing effect, making it more vulnerable to ROS attack and reduces the content of downstream metabolically pivotal compound. Besides, genes related to the biosynthesis of isoleucine, leucine, and valine were downregulated, and these three amino acids were essential for the growth of *S. aureus*, which represented an important group of nutrients for *S. aureus* metabolism and virulence [43]. The changes in the expression of these different genes may reduce the synthesis of key substances, making *S. aureus* less resistant to ROS and less virulent.

## 5. Conclusions

In the present study, we studied the effect of yellow light on the air microbiota in hospital wards, as well as the effect of yellow light on *E. coli*, *S. aureus*, and *P. aeruginosa*, three common hospital pathogens. We found that yellow light could reduce the abundance of air microorganisms in wards. In addition, by regulating the expression level of bacterial transcriptome, yellow light reduced the expression of virulence genes or metabolism genes in bacteria and promoted the death of bacteria. Our results provide meaningful information on the mechanism by which yellow light affects ward microbes.

## Data Availability

The datasets presented in this study can be found in online repositories. The names of the repository/repositories and accession number(s) can be found in the article.
